# Differences in Cellulosic Supramolecular Structure of Compositionally Similar Rice Straw Affect Biomass Metabolism by Paddy Soil Microbiota

**DOI:** 10.1371/journal.pone.0066919

**Published:** 2013-06-19

**Authors:** Tatsuki Ogura, Yasuhiro Date, Jun Kikuchi

**Affiliations:** 1 Graduate School of Medical Life Science, Yokohama City University, Yokohama, Kanagawa, Japan; 2 RIKEN Center for Sustainable Resource Science, Yokohama, Kanagawa, Japan; 3 Graduate School of Bioagricultural Sciences, Nagoya University, Nagoya, Aichi, Japan; 4 Biomass Engineering Program, RIKEN Research Cluster for Innovation, Wako, Saitama, Japan; J. Craig Venter Institute, United States of America

## Abstract

Because they are strong and stable, lignocellulosic supramolecular structures in plant cell walls are resistant to decomposition. However, they can be degraded and recycled by soil microbiota. Little is known about the biomass degradation profiles of complex microbiota based on differences in cellulosic supramolecular structures without compositional variations. Here, we characterized and evaluated the cellulosic supramolecular structures and composition of rice straw biomass processed under different milling conditions. We used a range of techniques including solid- and solution-state nuclear magnetic resonance (NMR) and Fourier transform infrared spectroscopy followed by thermodynamic and microbial degradability characterization using thermogravimetric analysis, solution-state NMR, and denaturing gradient gel electrophoresis. These measured data were further analyzed using an “ECOMICS” web-based toolkit. From the results, we found that physical pretreatment of rice straw alters the lignocellulosic supramolecular structure by cleaving significant molecular lignocellulose bonds. The transformation from crystalline to amorphous cellulose shifted the thermal degradation profiles to lower temperatures. In addition, pretreated rice straw samples developed different microbiota profiles with different metabolic dynamics during the biomass degradation process. This is the first report to comprehensively characterize the structure, composition, and thermal degradation and microbiota profiles using the ECOMICS toolkit. By revealing differences between lignocellulosic supramolecular structures of biomass processed under different milling conditions, our analysis revealed how the characteristic compositions of microbiota profiles develop in addition to their metabolic profiles and dynamics during biomass degradation.

## Introduction

Plant biomass is the most abundant and important material in the terrestrial biosphere. Its major components, namely, cellulose, hemicellulose, and lignin, are complex molecules that are abundantly produced in plant cell walls. Cellulose is a linear condensation polymer comprising β (1→4)-linked D-glucose units with a degree of polymerization ranging from 100 to 20,000 [1]. The strong interchain hydrogen bonding between the hydroxyl groups of adjacent cellulose polymers [2], [3] renders crystalline cellulose resistant to enzymatic hydrolysis [3]. Hemicelluloses are branched polymers, and their molecular masses are lower than those of cellulose [4]. The main constituents of hemicelluloses are glucose, mannose, galactose, xylose, and arabinose [4]. Lignins are reticulated, cross-linked macromolecules composed of phenylpropanoid units, which include *p*-hydroxyphenyl, guaiacyl, and syringyl [5]. Besides being the second most available biological polymer on Earth, lignin is exceptionally resistant to biodegradation [6], [7]. These three persistent components form a supramolecular structure, lignocellulose, in which cellulose and hemicellulose are cemented by lignin. Lignocellulosic cell walls play important roles in strengthening plants, protecting them against microbial attack, and increasing their survival chances in severe environments [7]–[9].

Lignocellulose has a strong and stable structure and is resistant to biodegradation. However, recent studies have shown that microbial communities mediate plant biomass degradation in animals, including detritivores, ruminants, termites, and omnivores [10], [11]. For example, gram-positive rumen bacterium *Ruminococcus albus* is widely recognized for its high cellulolytic activity [12]. *Thermobifida fusca*, a primary degrader of plant biomass in soil, as well as white-; brown-; and soft-rot fungi, hydrolyzes polysaccharides and lignin in lignocellulose to monosaccharides and aromatic rings [13]–[16]. The resulting monosaccharides are used for fermentation, and the remaining monosaccharides and aromatic rings are stored as humic substances in land environments [17]. However, the degradation profiles of complex microbiota in biomass with differences in cellulosic supramolecular structures but without compositional variations have yet to be elucidated.

To understand the cellulosic supramolecular structure and composition of biomass, persistent biomass samples must be pretreated using physical and/or chemical techniques such as milling, enzyme hydrolysis, heating, and ionic water extraction [18]–[21]. Ball-milled plant cell walls can be completely dissolved in various solvents such as 2N sodium hydroxide, 50% aqueous sodium thiocyanate, 60% aqueous lithium bromide, and formic acid [22]. A ball milling (BM) step is also required to isolate lignin from plant biomass. These pretreatment processes enable the analysis of persistent plant biomass using a combination of techniques such as solid-state nuclear magnetic resonance (NMR) [23]–[26], Fourier transform infrared (FTIR) spectroscopy [27], [28], X-ray diffraction for structural analysis [29], [30], solution-state NMR [31]–[34], gel permeation chromatography (GPC) [20], [35], and pyrolysis/gas chromatography-mass spectrometry (Py/GC-MS) [7], [36].

In our previous reports, we have performed unique studies using combined solid- and solution-state NMR and other methods in worldwide biomass experiments [37], [38]. Okushita et al. investigated structural changes in bacterial cellulose by subjecting ionic liquids to solid- and solution-state NMR and FTIR. The data were then profiled using principal components analysis (PCA) [39], [40]. Watanabe et al. profiled the tissue-specific biomass of *Jatropha curcas* using FTIR and solution-state NMR [41]. Date et al. described biochemical complexes in various seaweeds by the comprehensive characterization of water-soluble, water-insoluble, and solid-state components using FTIR and NMR [42].

Moreover, we developed a web-based toolkit, “ECOMICS,” for trans-omics analysis of ecosystems [43], [44]. The toolkit is free and includes several software tools; FT2DB, HetMap, Bm-Char, and E-class. FT2DB converts one-dimensional (1D)- and two dimensional (2D)-NMR spectra to digital data that can be statistically analyzed. The statistical analysis tool HetMap integrates and displays associations between heterogeneous datasets. Bm-Char can assign query chemical shifts to 88 known chemical signals of lignocellulose components, including 42 and 17 signals of aromatic and aliphatic sites in lignin, respectively, 26 of hemicellulose sites, and 3 of uncategorized sites, as previously reported [43]. The E-class analysis tool implements BLAST searches against numerous sequences stored in useful databases. Thus, the ECOMICS toolkit is a powerful and useful means of evaluating complex environmental samples such as plant biomass. It also enables the integration of multiple measurements of heterogeneous matrix data.

**Figure 1:**
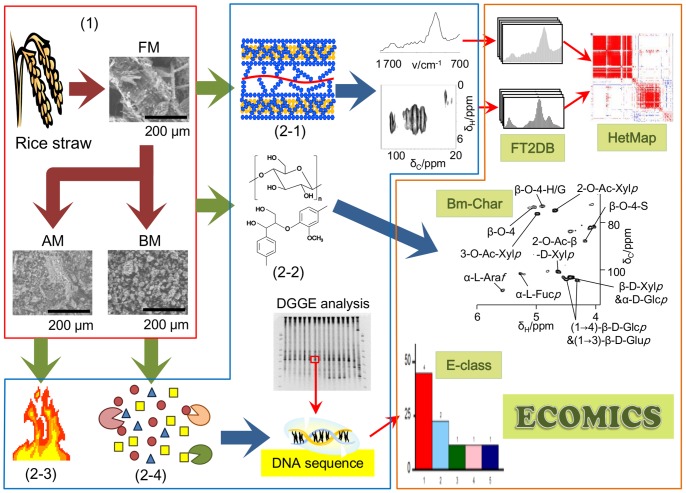
Schematic overview of this study. The effects of rice straw pretreatment on the cellulosic supramolecular structure and improvements in digestibility of lignocellulosic biomass for paddy soil microbiota were evaluated by physicochemical and biochemical methods. Rice straw samples were powdered using a blender, AM machine, and BM machine (1). Structural, compositional, thermodynamic, and degradability characterization (from 2–1 to 2–4) were performed using multimeasurement techniques such as FTIR, NMR, TG/DTA, and DGGE fingerprinting. Data were analyzed using the ECOMICS web-based toolkit.

This study focused on the effects of rice straw pretreatment on the cellulosic supramolecular structure and aimed to improve digestibility of lignocellulosic biomass for paddy soil microbiota. Supramolecular structures and composition of biomass were characterized using multiphysicochemical approaches combined with the ECOMICS web tools for multivariate data analysis (Fig. 1). Paddy soil microbiota was classified using E-class. Subsequently, the biomass degradation profiles of paddy soil microbiota were evaluated.

## Experimental Methods

### Preparation of biomass samples

Samples were prepared by processing five types of rice straw under different milling conditions. Lyophilized, food milling (FM)-processed rice straw samples were processed in a blender. Some FM-processed samples were further ground in an auto-milling (AM) machine (Tokken Inc., Chiba, Japan) and a planetary BM machine (Fritsch Japan Co., Ltd., Kanagawa, Japan) to produce AM- and BM-processed samples, respectively. AM-processed samples were prepared by grinding 300–400 mg of FM-processed samples in a stainless steel crusher of an AM machine operated at 1550 rpm for 3 min (AM_1_) or 10 min (AM_2_). BM-processed samples were prepared by placing approximately 300–400 mg of FM-processed samples in ZrO_2_ rotors (interior volume  = 12 ml) containing 50 ZrO_2_ balls (diameter  = 5 mm) and were then processed using a BM machine at 400 rpm for 1 h (BM_1_) or 6 h (BM_2_). Grinding was cyclically performed for 10 min and was then interrupted for the same length of time to prevent an excessive rise in sample temperature. Thus, the number of on/off cycles was set to 6 and 36 for BM_1_ and BM_2_ pretreatments, respectively.

### Scanning electron microscopy (SEM)

The cellulosic supramolecular structures of milling-processed rice straw samples were analyzed using a Hitachi TM-1000 scanning electron microscope (Hitachi High-Technologies Corp., Tokyo, Japan) with a backscattered electron (BSE) detector operating in the variable pressure (VP)-scanning electron microscopy (SEM) mode (column pressure retained at 30 Pa).

### Attenuated total reflectance (ATR)-FTIR analysis

Attenuated total reflectance (ATR)-FTIR provides information on structural changes in functional groups of rice straw after milling. ATR-FTIR spectra (4500–650 cm^−1^) were obtained using a Nicolet 6700 FTIR (Thermo Fisher Scientific Inc., Waltham, MA, USA) instrument with a KBr disk. The ATR smart iTR accessory with a high-pressure clamp (Thermo Fisher Scientific Inc.) was used. The spectra were obtained using triangular apodization with a resolution of 4 cm^−1^ and an interval of 1 cm^−1^. Each background and sample spectrum was obtained from 32 scans. The ONMIC software supplied with the equipment provided baseline and ATR corrections for penetration depth and frequency variations.

### Solid-state NMR

Solid-state NMR spectra were measured in a B0 field of 18.8 Tesla using a Bruker AV800 spectrometer (Rheinstetten, Germany; 800.20 MHz of ^1^H frequency) with a 54-mm narrow-bore magnet at room temperature (25±1°C). 1D cross-polarization-magic angle spinning (CP-MAS) and 2D ^13^C-^1^H heteronuclear correlation (HETCOR) spectra were obtained using a Bruker 4-mm double-tuned MAS probe. For the NMR measurements, approximately 80 mg of sample was placed in a ZrO_2_ rotor (outer diameter  = 4 mm) with a KelF-made cap. The MAS spinning speed, set to 12,000 Hz, was regulated using a Bruker MAS II pneumatic MAS controller. The contact time was set to 1.0 ms and 5.0 ms, and the recycle delay was 4 s. The MAS frequency was set to 12,000 Hz. The magic angle (54.7°) pulse length for protons was set to 1.8 μs. CP-MAS and cross-polarization-total sideband suppression (CP-TOSS) spectra were also measured using a Bruker DRX-500 spectrometer operating at 500.13 MHz for ^1^H equipped with the Bruker 4-mm double-tuned MAS probe to check the effect of spinning sidebands (SSB). The MAS spinning speed, set to 6000 Hz (for CP-MAS and CP-TOSS) and 12,000 Hz (for CP-MAS), was regulated using the Bruker MAS II pneumatic MAS controller with careful temperature controls.

### ^1^H-^13^C heteronuclear single quantum coherence (HSQC) NMR analysis for biomass samples

Sixty milligrams of each milled biomass sample were suspended in 1 ml of methanol. The mixture was heated at 50°C for 5 min in a Thermomixer comfort (Eppendorf AG, Hamburg, Germany) and then centrifuged. The supernatants were discarded, and the pellets were resuspended in methanol and centrifuged. The process was repeated twice more, giving a total of three suspensions. The pellets were then suspended in 1 ml of H_2_O and were processed three times as well as methanol extraction. After drying, the pellets were dissolved in 20 μl of a DMSO-*d*_6_/pyridine-*d*_5_ (4∶1) solvent per 1 mg of sample. After centrifugation, the supernatants containing the DMSO-*d*_6_/Pyridine-*d*_5_ (4∶1) solvent were used for NMR experiments. NMR spectra were acquired on a 700.15 MHz (AV700) Bruker Biospin instrument equipped with an inverse (^1^H coils closest to the sample) gradient 5-mm TBI ^1^H/^13^C/^15^N probe at 45°C. The central DMSO solvent peak was used as an internal reference (δ_C_  = 40.03, δ_H_  = 2.582 ppm). ^1^H-^13^C HSQC spectra were programed using a Bruker standard pulse sequence “hsqcetgp” (phase-sensitive gradient-edited-2D HSQC using trim pulses in INEPT transfer). NMR spectra were acquired from 14 to −3 ppm in F2 (^1^H) using 1024 data points for an acquisition time (AQ) of 60 ms, an interscan delay (D1) of 750 ms, and 179–9 ppm in F1 (^13^C) using 120 increments (F1 AQ  = 5.78 ms) of 340 scans. The obtained spectra were assigned by Bm-char (RIKEN database; https://database.riken.jp/ecomics/biomass/) [43], [44].

### Thermogravimetric/differential thermal analysis (TG/DTA)

Thermogravimetric (TG) analysis was conducted using an EXSTAR TG/differential thermal analysis (DTA) 6300 (SII Nanotechnology Inc., Tokyo, Japan) instrument. Approximately 9 mg of sample was individually loaded into an aluminum pan and vaporized (heating rate  = 5°C/min, from 25 to 500°C) in a nitrogen atmosphere with a flow rate of 200 ml/min. Based on the TG data, the thermodegradation kinetic parameters of milling-processed samples were analyzed using Coats and Redfern's integral method [45].

### Evaluation of biomass degradation profiles based on different milling conditions

A soil microbial community from a paddy field was used as seed material. The paddy soil sample was obtained from a private paddy field in Yamagata, Japan, with permission from the owner to conduct this study. To evaluate the biomass degradation profiles of soil microbiota under different milling conditions, 120 g of paddy soil was prepared and mixed with 480 ml of distilled water. Separate 80-ml aliquots of the mixture were poured into six 100-ml vials. Subsequently, 500 mg of biomass samples milled under different conditions (as described above) were added to each vial and incubated at 30°C with shaking at 160 rpm for 34 days. Incubated samples from each day were centrifuged to separate the supernatant from the pellet. The metabolic profiles of soil microbiota were evaluated from ^1^H- and ^1^H-^13^C HSQC NMR measurements of the supernatants, while the microbiota profiles were determined from denaturing gradient gel electrophoresis (DGGE) analysis of the pellets.

### ^1^H- and ^1^H-^13^C HSQC NMR measurements of metabolites produced during biomass degradation

The supernatant was prepared as described above, with slight modifications. All 1D Watergate spectra were acquired on a Bruker Avance DRX-500 NMR spectrometer operating at 500.13 MHz equipped with a 5-mm ^1^H inverse TXI probe with triple-axis gradients at 25°C [46], [47].

Metabolites assigned as acetate, butyrate, and propionate were quantified by calibration [48]. Standard acetate, butyrate, and propionate preparations (of known concentrations) were precisely quantified at three points under the same NMR measurement conditions. The concentrations of acetate, butyrate, and propionate in the samples were quantified by interpolating the standard curves.

To assign metabolites produced by soil microbiota, the supernatants from the first and second days of incubation with BM_2_-processed samples were measured using ^1^H-^13^C HSQC. NMR spectra were acquired on the 700.15 MHz (AV700) Bruker Biospin instrument equipped with an inverse (^1^H coils closest to the sample) gradient 5-mm Cryo ^1^H/^13^C/^15^N probe at 25°C. The acetate peak was used as an internal reference (δ_C_  = 66, δ_H_  = 1.9 ppm). NMR spectra were acquired from 10.192 to −0.788 ppm in F2 (^1^H) using 2048 data points for an AQ of 133 ms, D1 of 2 s, and 88–48 ppm in F1 (^13^C) using 142 increments (F1 AQ  = 9.07 ms) of 128 scans. The obtained spectra were assigned using SpinAssign (RIKEN database; http://prime.psc.riken.jp/) [49]–[51].

### PCR-DGGE and phylogenetic analysis

Microbial DNA extraction was performed using the PowerSoil^™^ DNA Isolation Kit (Mo Bio Laboratories Inc., Carlsbad, CA, USA) according to the manufacturer's instructions. The conditions and experimental procedures used for PCR amplification and DGGE performance have been reported elsewhere [48], [52]. The DGGE gels were stained with SYBR Green I (Lonza, Rockland, ME, USA) and were acquired using GelDoc XR (Bio-Rad laboratories Inc., Tokyo, Japan). To identify the bacterial origin of DNA sequences in the gel, selected DGGE bands were excised from the original gels, and their DNA fragments were amplified with corresponding primers as reported previously [48], [52]. The sequences were classified using the E-class tool (RIKEN database; https://database.riken.jp/ecomics/eclass/) [43], [44] and the Ribosomal Database Project (RDP; http://rdp.cme.msu.edu/) classifier [53], and related sequences were downloaded from the National Center for Biotechnology Information (NCBI). The sequences determined in this study and those retrieved from the databases were aligned using CLUSTAL W2 [54], [55]. A phylogenetic tree was constructed using CLUSTAL W2 and Genetyx-tree software by the neighbor-joining method [56].

### Statistical analysis

The ^1^H-NMR data were processed as ft2 files using NMRPipe software [57], [58]. To reduce the volume of data, spectra were subdivided into sequential 0.04 ppm designated regions between ^1^H chemical shifts of −0.5 and 9.5 using FT2DB (RIKEN database; https://database.riken.jp/ecomics/chika/index2.html), a web tool for digitizing 2D data [43], [44], [59]. After excluding water resonance, each region was integrated and normalized using the sum of the DSS integral regions [60]. ^13^C-^1^H HETCOR spectra were processed using NMRPipe software, and the ft2 files were digitized using FT2DB. Digital ^13^C-^1^H HETCOR data were corrected for ^13^C chemical shift, and data points consistent with the pseudo-^13^C CP-MAS NMR spectra were summed. The peak separations of ^13^C CP-MAS NMR spectra were performed using Fityk software (http://fityk.nieto.pl/) [61]. The DGGE image was analyzed using Quantity One software (Bio-Rad Laboratories Inc.). The signal intensities and band positions in each lane were divided into a spectrum of 100 variables.

PCA was performed according to our previous reports [42], [60] using R software. In brief, data were visualized as PCA score plots and loading plots. Each coordinate on the score plot represents an individual sample, whereas each coordinate on the loading plot represents a bacterially sourced DGGE band and a metabolite-sourced ^1^H-NMR spectral data point. Thus, the loading plots provide information on band positions or spectral regions responsible for the positions of coordinates or sample clusters in the corresponding score plots. A 2D correlation map of FTIR and ^13^C-^1^H HETCOR spectra was calculated as a symmetric matrix using the HetMap web tool for statistical analysis (RIKEN database; https://database.riken.jp/ecomics/chika/index.html) [43], [44].

## Results and Discussion

### Structural characterization of biomass processed under different milling conditions

To examine the lignocellulosic supramolecular structures of biomass processed under different milling conditions, the structures were observed using SEM (Fig. 1, SEM image). The structure of the FM-processed biomass sample was quite intact. In contrast, the BM_2_-processed sample appeared as a granulated powder, indicating structural breakage.

**Figure 2:**
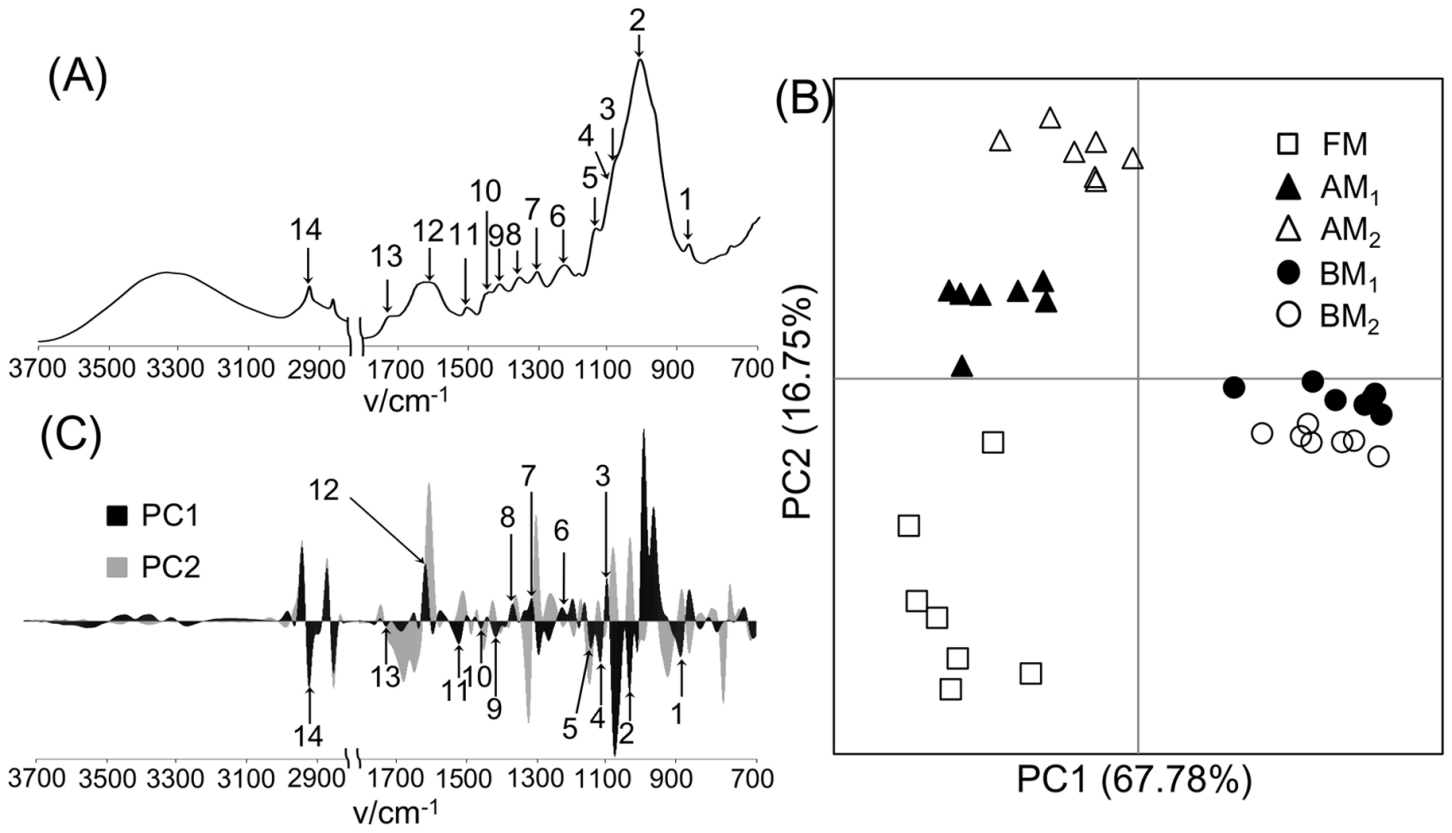
Structural characterization of biomass under different milling processes using FTIR spectroscopy. FTIR spectrum of the FM-processed sample (A), PCA score plot (B), and loading plot (C) of biomass degradation profiles based on FTIR spectra. 1, anomeric vibration at the β-glycosidic linkage; 2, C–O stretching in cellulose and hemicellulose; 3, vibration of ester linkage; 4, aromatic skeletal and C–O stretching; 5, deformation vibrations of C–H bonds in benzene rings; 6, syringyl ring and C–O stretching in lignin and xylan; 7, C–H in cellulose and C_1_–O vibration in a syringyl derivative; 8, C–H deformation in cellulose and hemicellulose; 9, aromatic ring vibrations; 10, asymmetric C–H bonding in CH_3_ and –CH_2_–; 11, aromatic ring vibrations; 12, aromatic ring vibrations and C = O stretching; 13, stretching of C = O unconjugated to aromatic rings (oxidized side chains); 14, C–H stretching. Open square, FM-; closed triangle, AM_1_-; open triangle, AM_2_-; closed circle, BM_1_-; open circle, BM_2_-processed samples.

Each milling-processed sample was also measured using ATR-FTIR (Fig. 2A). The characteristic peaks in ATR-FTIR spectra appeared at 890 cm^−1^ (anomeric vibration at β-glycosidic linkage), 1027 cm^−1^ (C–O stretching in cellulose and hemicellulose), 1103 cm^−1^ (vibration of ester linkage), 1120 cm^−1^ (aromatic skeletal and C–O stretching), 1145 cm^−1^ (deformation vibrations of C–H bonds in benzene rings), 1232 cm^−1^ (syringyl ring and C–O stretching in lignin and xylan), 1311 cm^−1^ (C–H in cellulose and C_1_–O vibration in syringyl derivatives), 1359 cm^−1^ (C–H deformation in cellulose and hemicellulose), 1413 cm^−1^ and 1502 cm^−1^ (aromatic ring vibrations), 1450 cm^−1^ (asymmetric C–H bonding in CH_3_ and –CH_2_–), 1604 cm^−1^ (aromatic ring vibrations and C = O stretching), 1720 cm^−1^ (stretching of C = O unconjugated to aromatic rings, oxidized side chains), and 2915 cm^−1^ (C–H stretching) [7], [30]. FTIR spectra were differentiated to first-derivative spectra and were digitized for PCA. The PCA score plot showed that milling-processed samples were clustered according to differences in pretreatment conditions. In particular, BM-processed samples were clearly separated from samples processed under the other milling conditions in the PC1 direction (Fig. 2B). On the loading plot, this separation resulted from the aforementioned characteristic peaks, suggesting that BM pretreatment deformed the chemical structures of cellulose, hemicellulose, and lignin (Fig. 2C). Next, ATR-FTIR spectra were subjected to homogeneous correlation analysis using HetMap (Fig. S1). The threshold of displayed correlation coefficients (*r* = 0.7) and number of samples (*n* = 5) in the HetMap analysis were chosen to obtain significant correlations between the detected characteristic peaks with the lowest percentage of noise. Positive or negative correlations were consistent with the characteristic peaks contributing to the same or opposite directions in the PCA loading plots, respectively. These results were consistent with the previously reported cleavage of the β-O-4 and α-O-4 linkages in lignin observed after BM pretreatment [62], increased aromatic ring vibrations and C = O stretching observed in BM-pretreated hardwood lignin consumed by clear wing borer [7], and IR band alteration observed in ball-milled lignin, induced by numerous stretching and vibrational modes [63].

**Figure 3:**
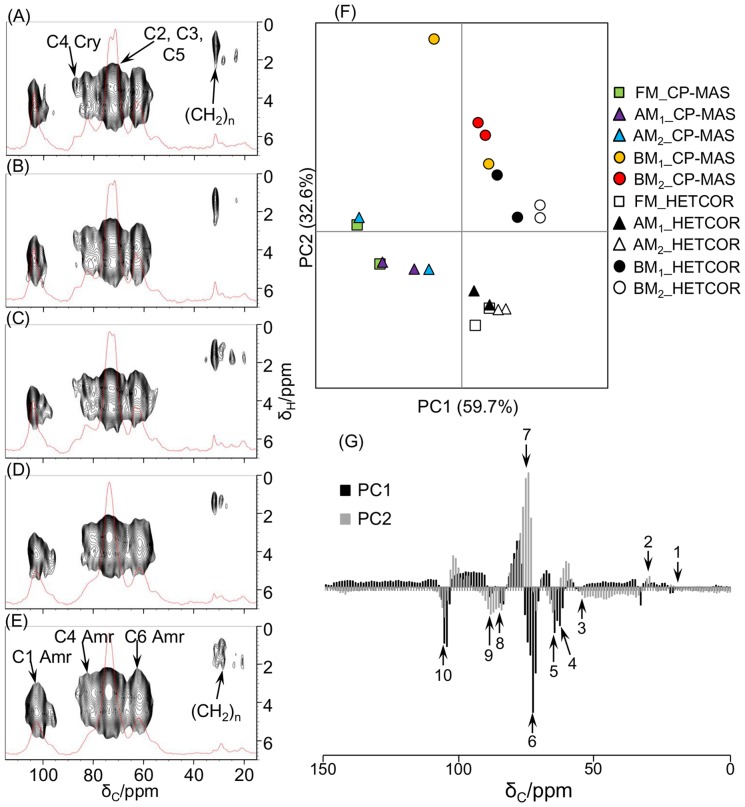
Structural characterization of biomass under different milling processes using NMR spectroscopy. ^13^C CP-MAS and ^13^C-^1^H HETCOR NMR spectra of FM- (A), AM_1_- (B), AM_2_- (C), BM_1_- (D), and BM_2_-processed samples (E). The contact time was set to 1.0 ms for CP-MAS spectra. Characteristics of the biomass structure of each sample: PCA score plot (F) and loading plot (G) of ^13^C CP-MAS and digitized 1D ^13^C-^1^H HETCOR spectra. C1–C6, position of cellulose carbon; cry, crystalline cellulose; amr, amorphous cellulose; 1, CH_3_ in hemicellulose; 2, aliphatic –(CH_2_)_n_–; 3, OCH_3_ of lignin; 4, CH_2_OH of carbohydrates (C6 of amorphous cellulose); 5, CH_2_OH of carbohydrates (C6 of crystalline cellulose); 6 and 7, CHOH of carbohydrates (C2, C3, and C5 of cellulose); 8, CHOH of carbohydrates (C4 of amorphous cellulose); 9, CHOH of carbohydrates (C4 of crystalline cellulose); 10, OCHO of carbohydrates (C1 of cellulose).

Similarly, ^13^C CP-MAS, CP-TOSS, and ^13^C-^1^H HETCOR NMR analyses were performed (Fig. 3 and Figs. S2 and S3). CP-MAS and CP-TOSS spectra were obtained using the FM-processed sample to optimize measurement conditions and to reduce the influence of aeolotropies and SSB. Small SSB signals (approximately 17, 26, 113, 121, and 153 ppm) were observed in the CP-MAS spectrum using the MAS spinning speed set to 6000 Hz compared with the CP-TOSS spectrum using the same spinning speed. Because the CP-TOSS spectrum had a lower resolution than the CP-MAS spectrum and biomass (cellulose) signals were less influenced by SSB signals in the CP-MAS spectrum using the spinning speed set to 12,000 Hz, the CP-MAS NMR spectrum measured using the spinning speed set to 12,000 Hz was used for further analysis.

The obtained CP-MAS and HETCOR spectra of milling-processed samples were assigned based on previous reports as follows: 21.5 ppm, CH_3_ of hemicellulose; 33 ppm, aliphatic –(CH_2_)_n_–; 63 ppm, CH_2_OH of carbohydrates (C6 of amorphous cellulose); 66 ppm, CH_2_OH of carbohydrates (C6 of crystalline cellulose); 72 ppm, CHOH of carbohydrates (C2, C3, and C5 of cellulose); 72–76 ppm, C–OR of lignin; 75 ppm, CHOH of carbohydrates (C2, C3, and C5 of cellulose); 84 ppm, CHOH of carbohydrates (C4 of amorphous cellulose); 89 ppm, CHOH of carbohydrates (C4 of crystalline cellulose); and 105 ppm, OCHO of carbohydrates (C1 of cellulose) [5], [64]. The C4 peak of crystalline cellulose was observed in the AM_1_- and AM_2_-processed biomass samples, as well as in the sample processed under the FM condition alone. However, a very small C4 peak of crystalline cellulose appeared in the BM_1_-processed sample but was absent in the BM_2_-processed sample, indicating physical fracture of the crystalline structure of biomass. This result was consistent with a previous report that peaks of 108 ppm and 60 ppm in ^13^C NMR spectra broadened after intense milling [65]. Moreover, multicomponent peaks obscured in solid-state NMR spectra, corresponding to C2, C3, and C5 of amorphous and crystalline cellulose, were clearly discriminated by peak separations of NMR spectra using Fityk software (Fig. S3). The peak separations in each milling-processed sample revealed that the intensity of peaks of amorphous cellulose was increased, whereas the intensity of those of crystalline and OCH_3_ of lignin was reduced after BM pretreatment. In addition, the PCA score plot showed that milling-processed samples were clustered according to differences between FM/AM and BM pretreatment (Fig. 3F). Clustering of BM-processed samples in the loading plot was primarily due to C2, C3, and C5 peaks of amorphous cellulose (Fig. 3G and Fig. S3). Homogeneous correlation analysis of NMR spectra was also consistent with fracturing of the crystalline structure of biomass after BM pretreatment (Fig. S4A). In particular, positive correlations were found between peaks of crystalline and crystalline cellulose or between those of amorphous and amorphous cellulose, and negative correlations were found between peaks of crystalline and amorphous cellulose. Reduced crystallinity of cellulose in samples subjected to BM processing has been reported previously [66], and the carbon peaks of cellulose revealed a shift from the crystalline to amorphous state [67]. In this experiment, the observed changes in peaks and correlations confirmed that the cellulose crystalline structure was fragmented and became amorphous. Heterogeneous correlation analysis of NMR and FTIR spectra (Fig. S4B and C) revealed positive correlations between OCH of lignin and vibration of ester linkage, CH_2_OH of carbohydrates (C6 of crystalline cellulose) and COC vibration, CH_2_OH of carbohydrates (C6 of crystalline cellulose) and asymmetric C–H bonding, CH_2_OH of carbohydrates (C6 of crystalline cellulose) and C–H stretching in cellulose, CHOH of carbohydrates (C4 of amorphous cellulose) and deformation vibrations of C–H bonds on benzene rings, CHOH of carbohydrates (C4 of amorphous cellulose) and aromatic ring vibration, CHOH of carbohydrates (C4 of amorphous cellulose) and asymmetric C–H bonding, CHOH of carbohydrates (C4 of amorphous cellulose) and C–H stretching in cellulose, CHOH of carbohydrates (C4 of crystalline cellulose) and deformation vibrations of C–H bonds on benzene rings, OCHO of carbohydrates (C1 of cellulose) and aromatic ring vibration, OCHO of carbohydrates (C1 of cellulose) and asymmetric C–H bonding, and OCHO of carbohydrates (C1 of cellulose) and C–H stretching in cellulose. Negative correlations were found between CH_2_OH of carbohydrates (C6 of crystalline cellulose) and C–O stretching in cellulose and hemicellulose, CHOH of carbohydrates (C2, C3, and C5 of cellulose) and vibration of ester linkage, CHOH of carbohydrates (C2, C3, and C5 of cellulose) and deformation vibrations of C–H bonds on benzene rings, CHOH of carbohydrates (C2, C3, and C5 of cellulose) and aromatic ring vibration, CHOH of carbohydrates (C4 of amorphous cellulose) and C–O stretching in cellulose and hemicellulose, CHOH of carbohydrates (C4 of crystalline cellulose) and C–O stretching in cellulose and hemicellulose, and OCHO of carbohydrates (C1 of cellulose) and C–O stretching in cellulose and hemicellulose. These results suggest that several peaks originating from lignocellulose were changed as the biomass structure was altered from crystalline to amorphous state after BM pretreatment.

### Compositional characterization of lignocellulosic biomass

**Figure 4:**
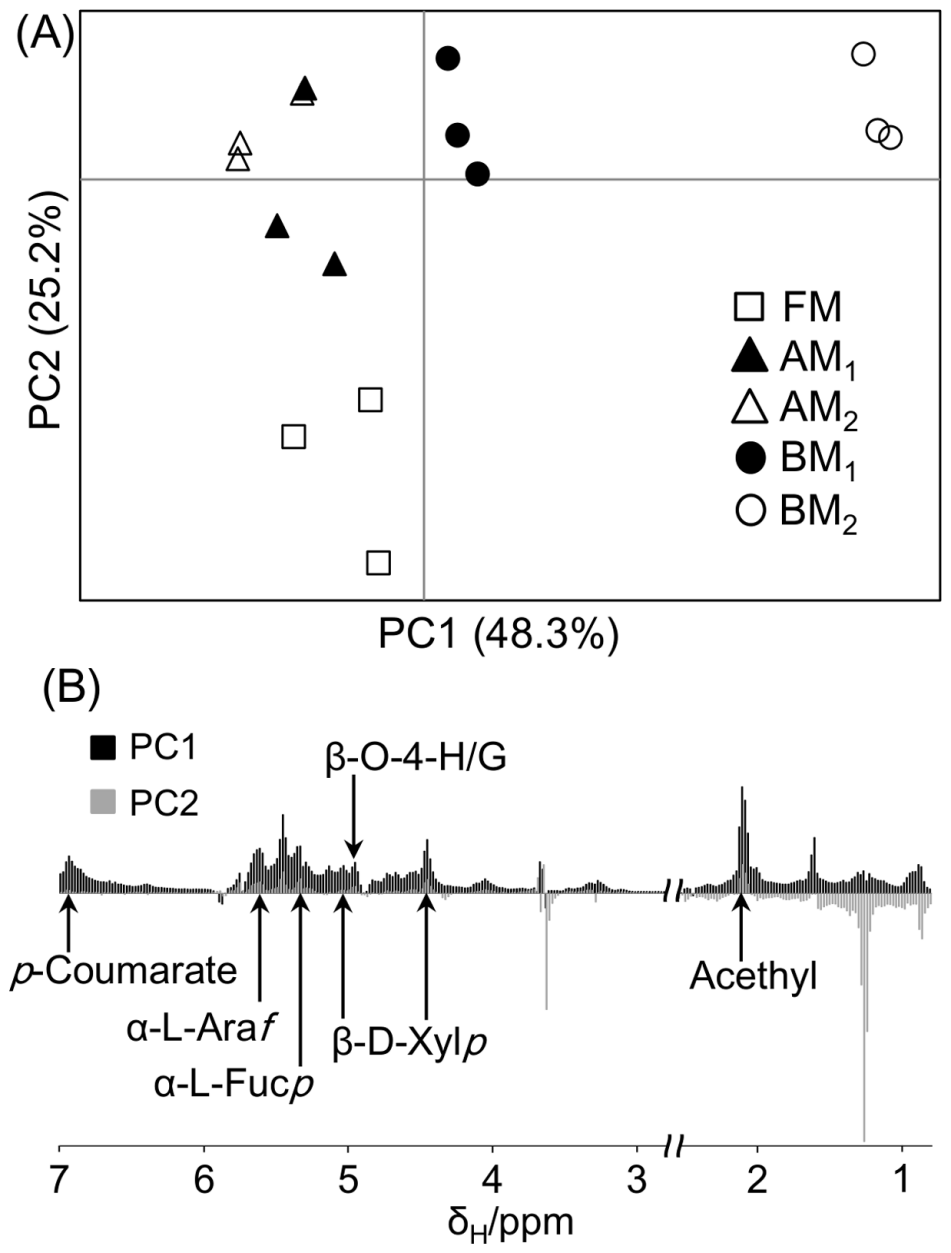
Compositional characterization of biomass under different milling processes using ^1^H-NMR spectroscopy. PCA score plot (A) and loading plot (B) of biomass degradation profiles based on ^1^H-NMR spectra of high-molecular-weight extracted components. The loading plot refers to ^1^H-^13^C HSQC spectra (see Fig. 5). Open square, FM-; closed triangle, AM_1_-; open triangle, AM_2_-; closed circle, BM_1_-; open circle, BM_2_-processed samples; β-D-Xyl*p*, β-D-xylopyranoside; α-L-Ara*f*, α-L-arabinofuranoside; α-L-Fuc*p*, α-L-fucopyranoside; G, guaiacyl; H, *p*-hydroxyphenyl; *p*CA, *p*-coumarate.

**Figure 5:**
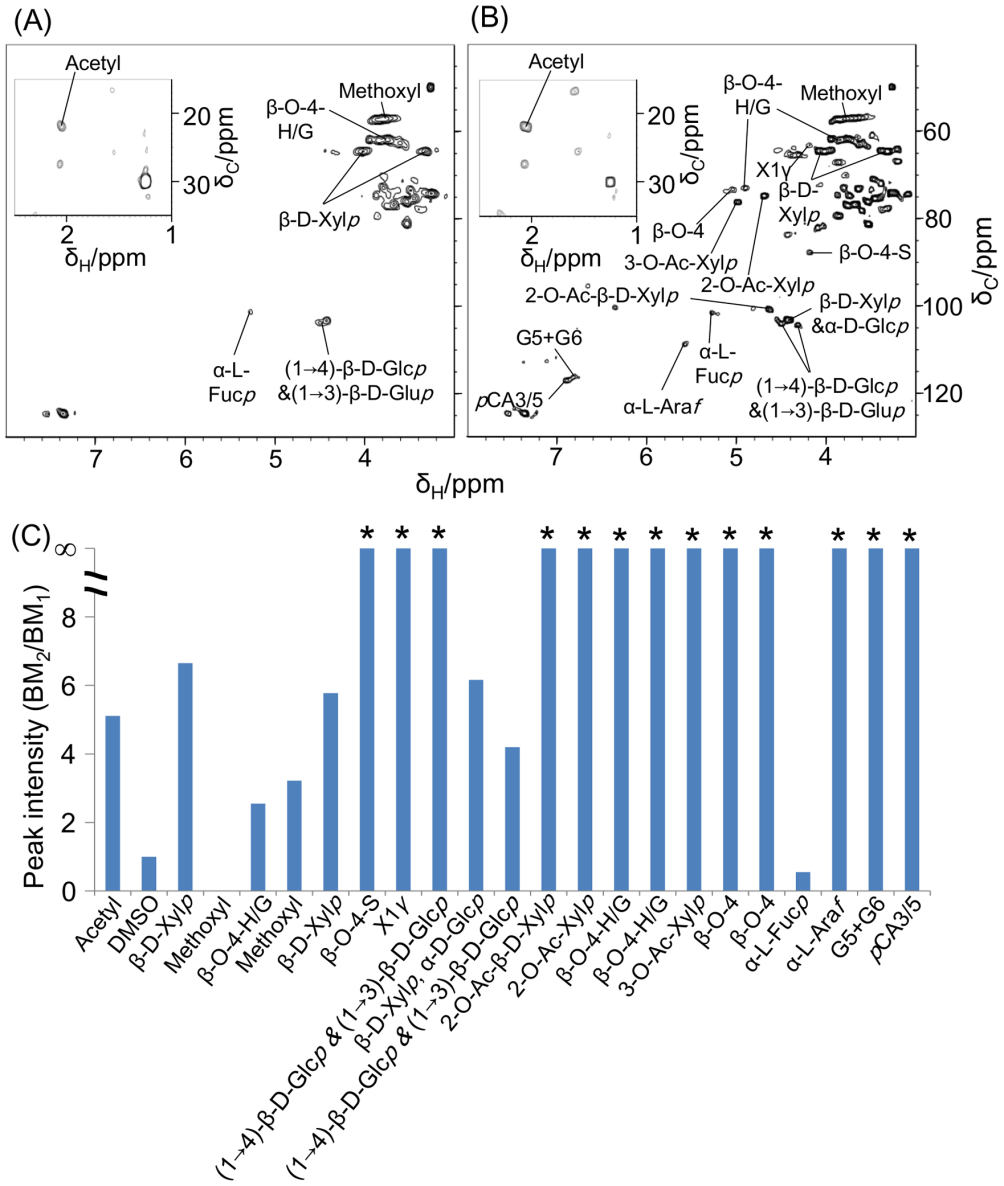
Compositional characterization of biomass observed in ^1^H-^13^C HSQC spectra. ^1^H-^13^C HSQC spectra of BM_1_- (A) and BM_2_-processed samples (B), and their ratios (BM_2_/BM_1_) of peak intensity (C). *, not detected in BM_1_-processed samples; α-D-Glc*p*, α-D-glucopyranoside; β-D-Glc*p*, β-D-glucopyranoside; β-D-Xyl*p*, β-D-xylopyranoside; α-L-Ara*f*, α-L-arabinofuranoside; α-L-Fuc*p*, α-L-fucopyranoside; 2-*O*-Ac-β-D-Xyl*p*, acetylated β-D-Xyl*p*; X1γ, γ-position of cinnamyl alcohol end group; S, syringyl; G, guaiacyl; H, *p*-hydroxyphenyl; *p*CA, *p*-coumarate.

^1^H- and ^1^H-^13^C HSQC spectra of biomass samples extracted using DMSO/pyridine were analyzed (Figs. 4 and 5). ^1^H-NMR spectral data were digitized using FT2DB and evaluated using PCA. AM-processed samples contributed to the positive direction of PC2, whereas BM-processed samples contributed to the positive directions of PC1 and PC2 (Fig. 4A). This separation was related to the low solubility of FM- and AM-processed samples in the DMSO/pyridine solvent compared with BM-processed samples (Fig. 4B). In addition, many peaks were annotated as lignocellulosic components such as acetyl (2.1 ppm), β-D-xylopyranoside (β-D-Xyl*p*; 4.46 ppm and 5.04 ppm), β-O-4-*p*-hydroxyphenyl/guaiacyl (β-O-4-H/G; 4.96 ppm), α-L-fucopyranoside (Fuc*p*; 5.36 ppm), α-L-arabinofuranoside (α-L-Ara*f*; 5.64 ppm), and *p*-coumarate (*p*CA; 6.94 ppm).

Because it was more soluble in DMSO/pyridine compared with samples pretreated under the other milling conditions, the BM_2_-processed sample displayed the highest number of peaks in its ^1^H-^13^C HSQC spectra (Fig. 5A and B). ^1^H-^13^C HSQC spectra of the DMSO/pyridine supernatant were assigned using Bm-Char. After comparing the spectra of BM_2_- and BM_1_-processed samples, many peaks were detected only in the BM_2_-processed sample, and the intensity of detected components in the BM_2_-processed sample was higher than that in the BM_1_-processed sample, except for the α-L-Fuc*p* signal (Fig. 5C)_._ This result suggests that increasing the time of BM processing from 1 to 6 h increased the proportion of extractable lignocellulosic components. Thus, cellulosic supramolecular structures of the BM_2_-processed sample were more fragmented than those of the BM_1_-processed sample. In a previous report, crystalline cellulose was converted to the amorphous form by vibratory BM [68]. In addition, BM pretreatment fragments lignin polymers *via* cleavage of β-ether bonds, with trivial development of carbonyl structures [62]. Similarly, in this study, BM pretreatment reduced the major insoluble components of lignocellulose to lower molecular mass molecules by significant bond cleavage. Consequently, BM-processed samples were relatively soluble in the DMSO/pyridine solvent. These results are largely consistent with the solid-state NMR and ATR-FTIR analyses.

### Thermodynamic characterization using TG/DTG analysis

**Figure 6:**
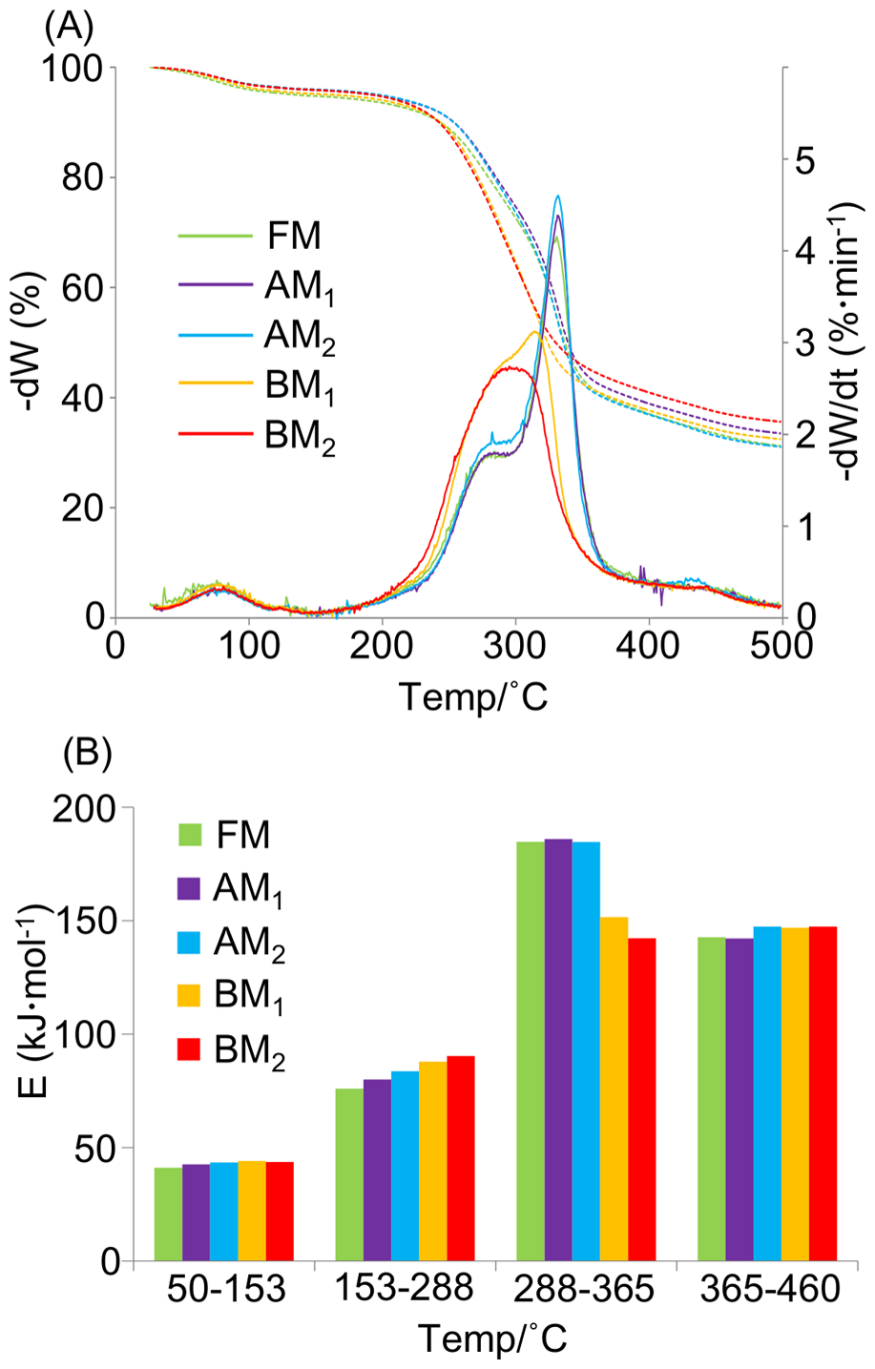
Thermodynamic characterization of biomass under different milling processes observed in TG/DTG analysis. TG and DTG degradation curves (A) and bar graph of the activation energy required to decompose lignocellulosic components (B) in samples subjected to different milling processes. Dashed lines, TG profiles; solid lines, DTG profiles.

The thermodynamic properties of biomass samples processed under different milling conditions were characterized by TG/DTG analysis (Fig. 6). DTG spectra of FM- and AM-processed samples displayed two main peaks at 279°C and 330°C, associated with xylan and cellulose, respectively. In contrast, the peaks of BM_1_- and BM_2_-processed samples were shifted downwards at 292°C and 318°C in BM_1_- and 295°C in BM_2_-processed samples (Fig. 6A). Therefore, the thermal degradation profiles of BM-processed samples were shifted to a lower temperature than those of FM- and AM-processed samples.

The activation energy of the thermal degradation profiles was determined using Coats and Redfern's integral model, as previously reported [7], [69]. The calculated activation energy ranges were observed at 50–153°C (dehydration), 153–288°C (mainly hemicellulose decomposition), 288–365°C (mainly cellulose decomposition), and 365–460°C (mainly lignin decomposition) (Fig. 6B). The activation energies in the regions of dehydration, hemicellulose decomposition, and lignin decomposition in all samples milled under different conditions were approximately 73, 74, and 125 kJ mol^−1^, respectively. However, the activation energies of the cellulose decomposition region varied among the samples. In FM- and AM-processed samples, this energy was approximately 166 Kj mol^−1^ but reduced to 110 kJ mol^−1^ and 96 kJ mol^−1^ in the BM_1_- and BM_2_-processed samples, respectively. This difference in the activation energy between the BM- and FM/AM-processed samples is attributable to marked changes in biomass structure as the cellulose degrades from crystalline to amorphous state after BM pretreatment.

### Degradability characterization of lignocellulosic biomass by paddy soil microbiota

**Figure 7:**
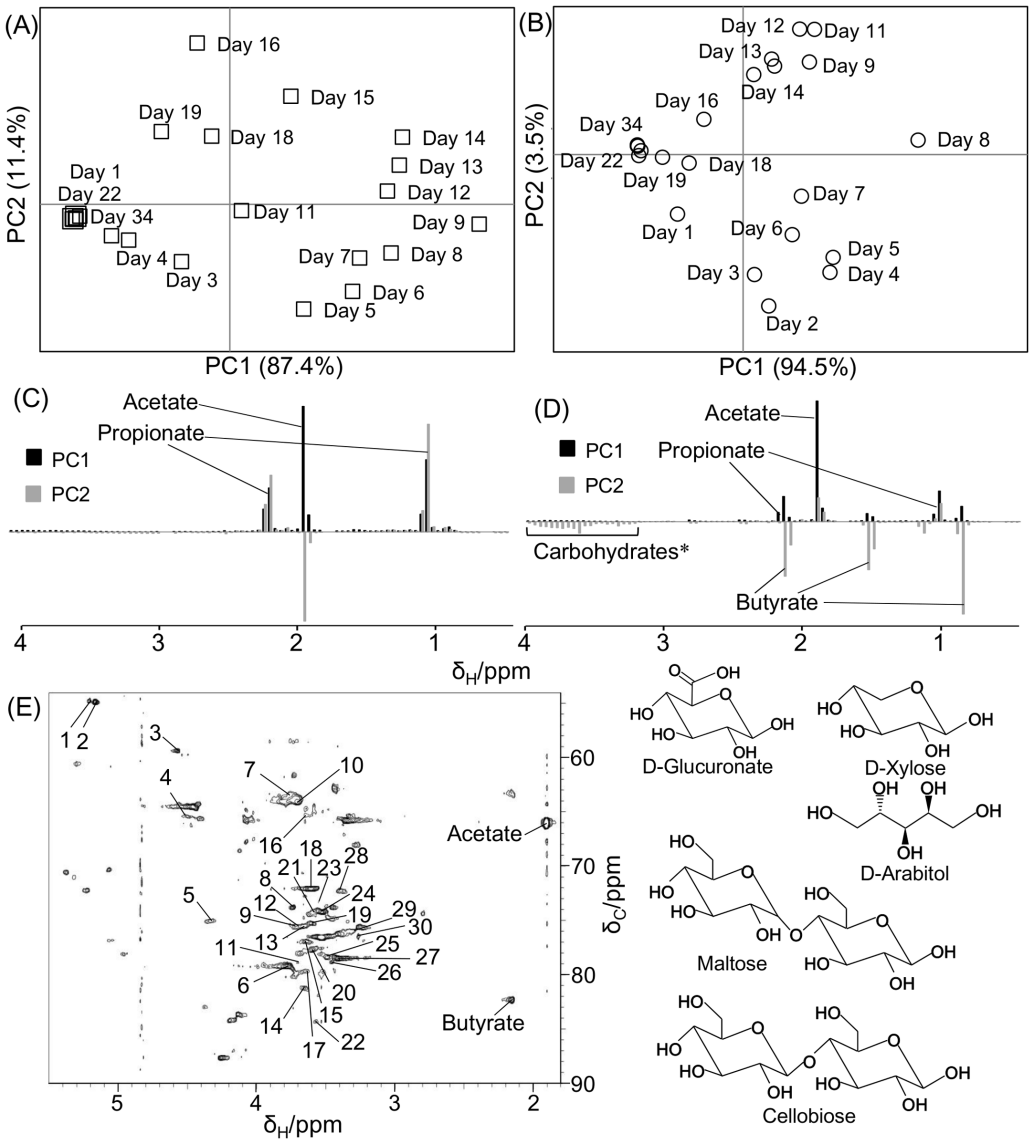
Degradability characterization observed in NMR analysis. Metabolic profiles of FM- (A, C) and BM_2_-processed samples (B, D) biomass samples evaluated using the PCA score plots (A, B) and loading plots (C, D) during biomass degradation by soil microbiota. Degraded biomass, including carbohydrates (*), in the BM_2_-processed sample is assigned from the ^1^H-^13^C HSQC NMR spectrum (E). The peaks indicated by numbers (listed in Table 1) were assigned as D-glucuronate, D-xylose, D-arabitol, cellobiose, and maltose.

To evaluate the effect of milling conditions on cellulosic supramolecular structure of biomass, each pretreated sample was incubated with paddy soil. Metabolites produced by soil microbiota were measured in the supernatant of the incubated samples by ^1^H-NMR spectroscopy. Few signals were observed in the control (no addition of the sample) compared with rice straw-incubated samples (Fig. S5); thus, the NMR signals of metabolites produced by microbiota and background noise from soil organics were easily distinguishable. NMR spectral data were then digitized and analyzed by PCA (Fig. 7A–D and Fig. S6). The daily changes in metabolites throughout biomass degradation are revealed in the PCA score plots of the FM- and BM_2_-processed samples (see Fig. 7A and B). The metabolic profiles in both samples varied sequentially from the first to the final day of incubation, finally converging at the first point in the PCA score plots. This convergence suggested that paddy soil microbiota had terminated the biomass degradation and metabolite production processes. In addition, microbiota in the BM_2_-processed sample began degrading biomass earlier than those in the FM-processed sample, suggesting that BM pretreatment improves the accessibility of soil microbiota to the lignocellulosic supramolecular structures.

Metabolites produced by the varying microbiota in the FM- and BM_2_-processed samples are shown in the loading plots in Fig. 7C and D. The FM-processed sample contributed acetate (1.92 ppm) and propionate (1.04 ppm and 2.16 ppm), whereas the BM_2_-processed sample contributed acetate, propionate, butyrate (0.88 ppm, 1.56 ppm, and 2.16 ppm), and sugar regions (from 3.2 to 4 ppm). Acetate and propionate contributed to PC1 and PC2 in the FM-processed sample and to PC1 in the BM_2_-processed sample. However, butyrate contributed to PC2 in the BM_2_-processed sample. These metabolites (i.e., acetate, butyrate, and propionate) were quantified from ^1^H-NMR spectral data (Fig. S7). Production of acetate and propionate was maximized at around Day 8, whereas that of butyrate was maximized at around Day 5. The BM_2_-processed sample ceased production of all three metabolites after approximately 21 days. In contrast, the FM-processed sample produced the highest concentrations of acetate and propionate at around Day 9, with metabolism ceasing at around Day 21. This sample produced very little butyrate. The total acetate concentrations produced in BM_2_- and FM-processed samples were approximately 400 mmol l^−1^ and 150 mmol l^−1^, respectively. Similarly, in BM_2_- and FM-processed samples, the total propionate concentrations were approximately 45 mmol l^−1^ and 37 mmol l^−1^, respectively, whereas those of butyrate were approximately 180 mmol l^−1^ and 50 mmol l^−1^, respectively. Overall, higher production of acetate, butyrate, and propionate were observed in the BM_2_-processed sample compared with the FM-processed sample, and the metabolic profiles differed significantly among the samples. Therefore, we can infer that the metabolic profiles of the biomass-degrading soil microbiota are affected by the altered biomass supramolecular structures of samples milled under different conditions.

To determine the unassigned signals, particularly in the sugar regions of ^1^H-NMR spectra, the signals in the BM_2_-processed sample were measured using the ^1^H-^13^C HSQC method and were assigned using the SpinAssign program (Fig. 7E, Table 1, and Table S1). In this analysis, the signals were annotated as cellobiose, D-arabitol, D-glucuronate, D-xylose, and maltose. The signals of these components decreased as biomass degradation by soil microbiota increased during early degradation. Therefore, the degradation of these components appears to be affected by the production processes of metabolites such as acetate, butyrate, and propionate. These components might directly affect butyrate production by soil microbiota, particularly given that butyrate was more rapidly produced in BM_2_- than in the FM-processed samples. Considering the structural, compositional, and thermodynamic data, the annotated sugars were likely derived from the constituent sugars in lignocellulose, because the lignocellulosic supramolecular structures of the BM_2_-processed sample had been physically fragmented after BM pretreatment, causing drastic changes in chemical structure, as described above.

**Table 1:** Annotated peaks of BM_2_-processed samples extracted using a D_2_O solvent that were detected in ^1^H-^13^C HSQC spectra.

	Chemical shift (ppm)		
Peak No.	^1^H	^13^C	Components
1	5.203	54.748	Cellobiose, D-Glucuronate
2	5.16	54.874	D-Xylose
3	4.565	59.361	D-Xylose
4	4.509	65.225	Cellobiose
5	4.314	74.971	Maltose
6	3.766	79.19	Maltose
7	3.761	63.367	Cellobiose, Maltose
8	3.73	73.766	D-Arabitol
9	3.713	75.508	D-Glucuronate, Maltose
10	3.697	64.072	D-Xylose
11	3.689	78.83	D-Glucuronate, Maltose
12	3.687	75.508	Maltose
13	3.654	75.459	D-Xylose, Maltose
14	3.648	81.166	Cellobiose
15	3.639	76.848	Cellobiose
16	3.638	65.248	D-Arabitol, Ethylene glycol
17	3.622	79.596	Maltose
18	3.599	72.023	D-Xylose
19	3.593	75.189	Maltose
20	3.586	77.645	Cellobiose, Maltose
21	3.579	74.355	Cellobiose, D-Glucuronate, Maltose
22	3.567	84.228	Glycine
23	3.547	73.259	D-Arabitol
24	3.52	74.192	D-Glucuronate, D-Xylose, Maltose
25	3.483	78.296	Cellobiose, D-Glucuronate
26	3.45	78.767	Cellobiose
27	3.397	78.434	D-Xylose
28	3.39	72.213	Cellobiose, Maltose
29	3.294	75.888	Cellobiose
30	3.258	76.402	Cellobiose, D-Glucuronate, Maltose

The microbiota profiles developed during the biomass degradation processes were evaluated by DGGE. Variations in the profiles of biomass-degrading microbiota in FM- and BM_2_-processed samples are shown in the PCA score plots (Fig. S8A). The microbiota profiles at Days 1–3 in both the samples were almost identical, as shown in the PCA score plots. During incubation, the microbiota profiles gradually diverged as biomass degradation progressed, as shown in the PCA score plot. Eventually, the profiles separated according to the metabolic differences between the FM- and BM_2_-processed samples. The sequenced DGGE bands are shown in the loading plot (Fig. S8B). DGGE Bands 1, 2, 3, 4, and 7 contributed to the clustering of the microbiota profiles in the FM-processed sample, whereas Bands 5, 6, 8, and 9 contributed to the clustering of the microbiota profiles in the BM_2_-processed sample. This result suggests that microbiota contributing to the profiles in FM- or BM_2_-processed samples account for the principal difference in biomass degradation between the two samples.

The microbiota detected in the DGGE analysis was identified by sequencing and classification, and phylogenetic analysis was performed using RDP classifier and E-class (Figs. S9 and S10, and Table 2). The bacterial sources of Bands 1, 2, 3, 7, and 9 belong to the phylum Proteobacteria; Bands 4, 5, and 6 to the phylum Bacteroidetes; and Band 8 to the phylum Chlorobi. These results, as determined by E-class [43], [44], were validated by the RDP classifier (http://rdp.cme.msu.edu/classifier/classifier.jsp) (Fig. S9). The closest relatives of the sequences derived from Bands 1, 2, 3, and 7 were *Acinetobacter brisouii* [DQ832256] (99% identity) and from Bands 6, 8, and 9 were *Paludibacter propionicigenes* [AB078842] (94% identity), *Ignavibacterium album* [AB478415] (93% identity), and *Azotobacter chroococcum* [EF634034] (100% identity), respectively. *P. propionicigenes* is an anaerobic bacterium associated with the degradation of rice plant residues in Japanese paddy soil [70]. This bacterium utilizes hemicellulose-derived components such as xylose, cellobiose, and maltose as growth substrates and produces acetate and propionate [71]. *I. album* was isolated from hot spring water streams. This organism utilizes D-glucose, D-mannose, D-fructose, maltose, and cellobiose [72], which are the main components of plant cell walls. Bacteria related to both these species, which belong to the phylum Bacteroidetes, were detected in the DGGE analysis. These organisms gradually dominated the biomass degradation process in the BM_2_-processed sample. Fragmentation of the lignocellulosic supramolecular structures of the BM_2_-processed sample may have enabled these bacteria to utilize the constituent sugars in lignocelluloses to produce the acetate, butyrate, and propionate observed after BM_2_ pretreatment.

**Table 2:** Taxonomic classification of the detected DGGE bands.

DGGE Band	Phylum	Class	Order	Family	Genus	Closest relative	Identification (%)
1	Proteobacteria	Gammaproteoba cteria	Pseudomonadales	Moraxellaceae	*Acinetobacter*	DQ832256	99
2	Proteobacteria	Gammaproteoba cteria	Pseudomonadales	Moraxellaceae	*Acinetobacter*	DQ832256	99
3	Proteobacteria	Gammaproteoba cteria	Pseudomonadales	Moraxellaceae	*Acinetobacter*	DQ832256	99
4	Bacteroidetes	Unclassified_Ba cteroidetes	-	-	-	AB443948	84
5	Bacteroidetes	Unclassified_Ba cteroidetes	-	-	-	AB443948	84
6	Bacteroidetes	Bacteroidia	Bacteroidales	Porphyromonadaceae	*Paludibacter*	AB078842	94
7	Proteobacteria	Gammaproteoba cteria	Pseudomonadales	Moraxellaceae	*Acinetobacter*	DQ832256	99
8	Chlorobi	Ignavibacteria	Ignavibacteriales	Ignavibacteriaceae	*Ignavibacterium*	AB478415	93
9	Proteobacteria	Gammaproteoba cteria	Pseudomonadales	Pseudomonadaceae	*Azotobacter*	EF634034	100

This study investigated the effects of rice straw pretreatment on the cellulosic supramolecular structure. The results will assist in improving digestibility of lignocellulosic biomass for paddy soil microbiota. The effects were successfully evaluated and characterized using the ECOMICS web-based toolkit. This toolkit enables the evaluation of biomass structures and components and integration of heterogeneous matrix data in environmental and metabolic systems. The ECOMICS toolkit revealed that physical pretreatment of rice straw alters the cellulosic supramolecular structure. Thermal degradation profiles were shifted to lower temperatures, and different microbiota profiles with different metabolic dynamics were evolved during the biomass degradation process.

## Conclusions

In this study, the cellulosic supramolecular structure of plant biomass was altered using physical milling processes, and as a result, biomass degradation by paddy soil microbiota was affected. The cellulosic supramolecular structures of biomass samples processed under different milling conditions showed distinct conformational differences; for instance, the cellulose structure was altered from crystalline to amorphous state. Extractable lignocellulosic components were degraded to lower molecular mass molecules after 6 h of BM pretreatment. TG/DTG analysis suggested that the activation energies of biomass decomposition were affected by the cellulose conformation. In addition, biomass samples with differences in cellulosic supramolecular structures, although without compositional variations, produced different metabolites on degradation and had characteristic paddy soil microbiota profiles. Degradation profiles of FM- and BM_2_-processed samples revealed differences in the types and quantities of metabolites produced. Further, in this study, the soil microbiota involved in the degradation of structurally different biomass samples were different from those generally associated with degradation. These evaluations and characterizations were successfully achieved using the ECOMICS toolkit.

## Supporting Information

Figure S1 **Homogeneous correlation analysis of ATR-FTIR using HetMap.** The list of 2D heat maps (A) is provided for comparison of differences between the number of samples (from 7 to 1) and thresholds (from 0.5 to 0.9). The 2D heat map at *n* = 5 and *r* = 0.7 is displayed (B). 1, COC vibration; 2, C–O stretching in cellulose and hemicellulose; 3, vibration of ester linkage; 4, aromatic skeletal and C–O stretching; 5, deformation vibrations of C–H bonds on benzene rings; 6, syringyl ring and C–O stretching in lignin and xylan; 7, C–H in cellulose and C_1_–O vibration in syringyl derivatives; 8, C–H deformation in cellulose and hemicellulose; 9, aromatic ring vibration; 10, asymmetric C–H bonding (in CH_3_ and –CH_2_–); 11, aromatic ring vibration; 12, stretching of C = O conjugated to aromatic rings; 13, stretching of C = O unconjugated to aromatic rings (oxidized side chains); 14, C–H stretching in cellulose.
(TIF)

Figure S2 **Comparisons of CP-MAS and CP-TOSS spectra using the spinning speed set to 6000 Hz and 12,000 Hz.** CP-MAS spectra measured using the spinning speed set to 6000 Hz (black) and 12,000 Hz (blue) and CP-TOSS spectra measured using the spinning speed set to 6000 Hz (red) were obtained using the FM-processed sample.
(TIF)

Figure S3 **Peak separation of solid-state NMR using Fityk software.** (A) FM-, (B) AM_1_-, (C) AM_2_-, (D) BM_1_-, and (E) BM_2_-processed samples.
(TIF)

Figure S4 **Homogeneous and heterogeneous correlation analysis of ^13^C-^1^H HETCOR and ATR-FTIR spectra.** Homogeneous correlation heat map of NMR spectra (A) and heterogeneous correlation heat maps calculated by Pearson (B) and Spearman (C) between ^13^C-^1^H HETCOR and ATR-FTIR spectra. 1, CH_3_ in hemicellulose; 2, aliphatic –(CH_2_)_n_–; 3, OCH_3_ of lignin; 4, CH_2_OH of carbohydrates (C6 of amorphous cellulose); 5, CH_2_OH of carbohydrates (C6 of crystalline cellulose); 6 and 7, CHOH of carbohydrates (C2, C3, and C5 of cellulose); 8, CHOH of carbohydrates (C4 of amorphous cellulose); 9, CHOH of carbohydrates (C4 of crystalline cellulose); 10, OCHO of carbohydrates (C1 of cellulose); 11, COC vibration; 12, C–O stretching in cellulose and hemicellulose; 13, vibration of ester linkage; 14, aromatic skeletal and C–O stretching; 15, deformation vibrations of C–H bonds on benzene rings; 16, syringyl ring and C–O stretching in lignin and xylan; 17, C–H in cellulose and C_1_–O vibration in syringyl derivatives; 18, C–H deformation in cellulose and hemicellulose; 19, aromatic ring vibration; 20, asymmetric C–H bonding (in CH_3_ and –CH_2_–); 21, aromatic ring vibration; 22, stretching of C = O conjugated to aromatic rings; 23, stretching of C = O unconjugated to aromatic rings (oxidized side chains); 24, C–H stretching in cellulose.
(TIF)

Figure S5 **Comparison of ^1^H-NMR spectra of the control and FM- or BM_2_-processed incubation samples at Day 8.** Red spectra are control and black spectra are FM- (A) and BM_2_-processed samples (B).
(TIF)

Figure S6 **Degradability characterization of metabolic profiles based on ^1^H-NMR spectra.** AM_1_- (A, B), AM_2_- (C, D), and BM_1_-processed (E, F) biomass samples were evaluated by the PCA score plots (A, C, and E) and loading plots (B, D, and F) of metabolic profiles during biomass degradation by soil microbiota.
(TIF)

Figure S7 **Time course variations of quantified metabolites based on ^1^H-NMR spectra.** Acetate (A: 1.92 ppm), propionate (B: 1.04 ppm), and butyrate (C: 0.88 ppm) were produced during biomass degradation by soil microbiota. Shown are the production quantity at measured times (open symbols) and their summations (closed symbols) in FM- (square symbols) and BM_2_-processed (circle symbols) samples.
(TIF)

Figure S8 **Degradability characterization of microbial community profiles based on DGGE fingerprinting.** Microbiota profiles during biomass degradation in FM- (open square) and BM_2_-processed (open circle) biomass samples were evaluated by the PCA score plots (A) and loading plots (B). Each number (Days 1–19) in the score plots indicates the day of incubation. Detailed informations on the detected bands are shown in Figs. S9 and S10 and summarized in Table 2.
(TIF)

Figure S9 **Phylogenetic tree constructed based on partial 16S rRNA gene sequences.** The sequences determined in this study and those retrieved from the databases were aligned using CLUSTAL W2. The phylogenetic tree was then constructed using CLUSTAL W2 and Genetyx-tree software by the neighbor-joining method. The 16S rRNA gene fragment was amplified using the Univ954f and Univ1369r primer sets. The clones obtained were expressed as DGGE Bands 1–9. The 16S rRNA gene sequence of *Aquifex pyrophilus* [M83548] was used as an outgroup to root the tree. Indicated numbers in phylogenetic tree are bootstrap values.
(TIF)

Figure S10 **Classification of DGGE band sequences using E-class.** Classification of each DGGE band sequence to the phylum (A) and genus level (B) and its summary (C).
(TIF)

Table S1 **The list of annotated metabolites in BM_2_ samples extracted by D_2_O solvent detected in the ^1^H-^13^C HSQC spectra.**
(DOCX)
