# Supplementary figures and images for: Differences in Cellulosic Supramolecular Structure of Compositionally Similar Rice Straw Affect Biomass Metabolism by Paddy Soil Microbiota
Source: PLoS One. 2013 Jun 19;8(6):e66919. doi: 10.1371/journal.pone.0066919 (PMC3686774; doi:10.1371/journal.pone.0066919)

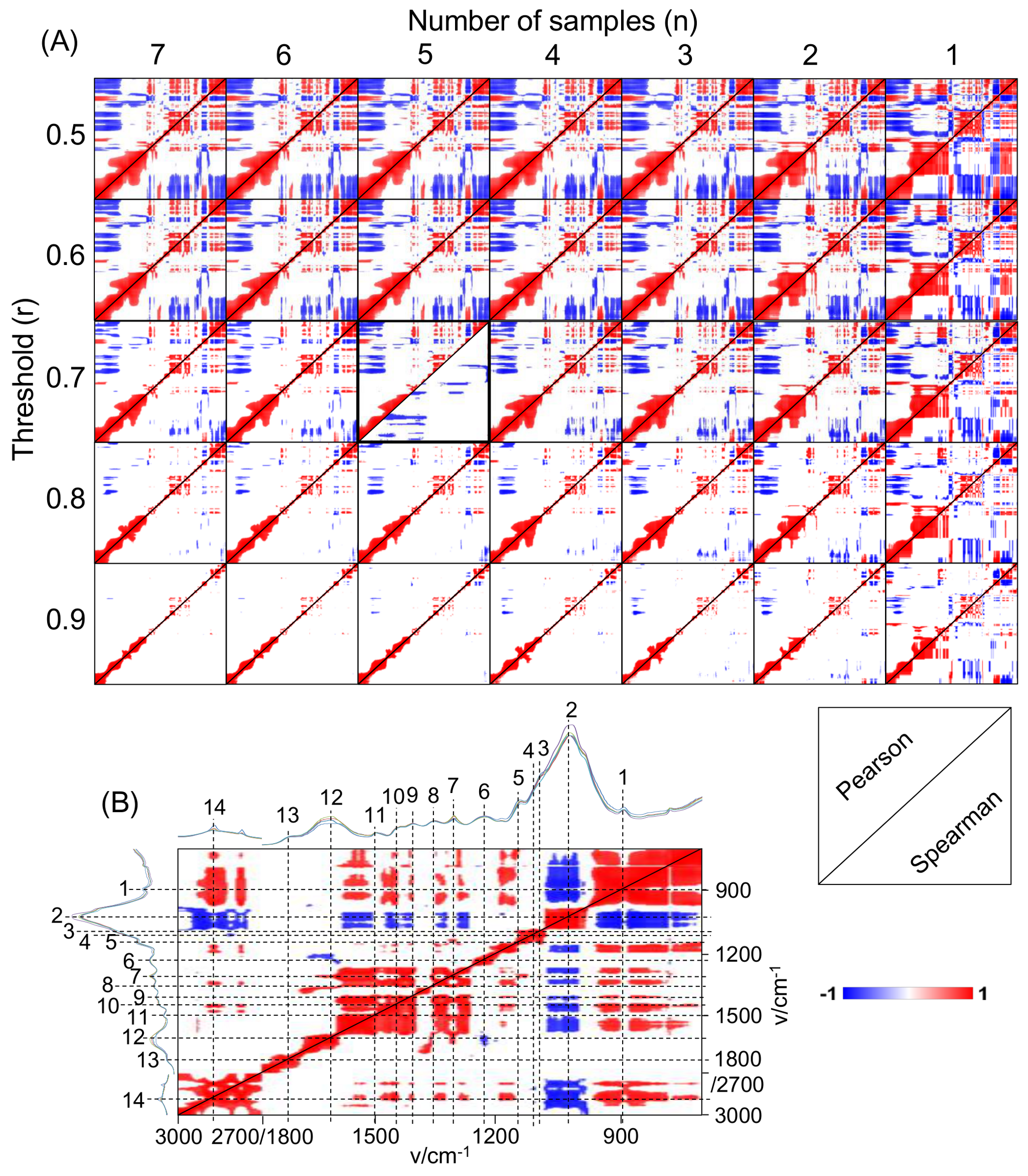

Supplement: Figure S1 — Homogeneous correlation analysis of ATR-FTIR using HetMap. The list of 2D heat maps (A) is provided for comparison of differences between the number of samples (from 7 to 1) and thresholds (from 0.5 to 0.9). The 2D heat map at n = 5 and r = 0.7 is displayed (B). 1, COC vibration; 2, C–O stretching in cellulose and hemicellulose; 3, vibration of ester linkage; 4, aromatic skeletal and C–O stretching; 5, deformation vibrations of C–H bonds on benzene rings; 6, syringyl ring and C–O stretching in lignin and xylan; 7, C–H in cellulose and C1–O vibration in syringyl derivatives; 8, C–H deformation in cellulose and hemicellulose; 9, aromatic ring vibration; 10, asymmetric C–H bonding (in CH3 and –CH2–); 11, aromatic ring vibration; 12, stretching of C = O conjugated to aromatic rings; 13, stretching of C = O unconjugated to aromatic rings (oxidized side chains); 14, C–H stretching in cellulose. (TIF) [file pone.0066919.s001.tif]

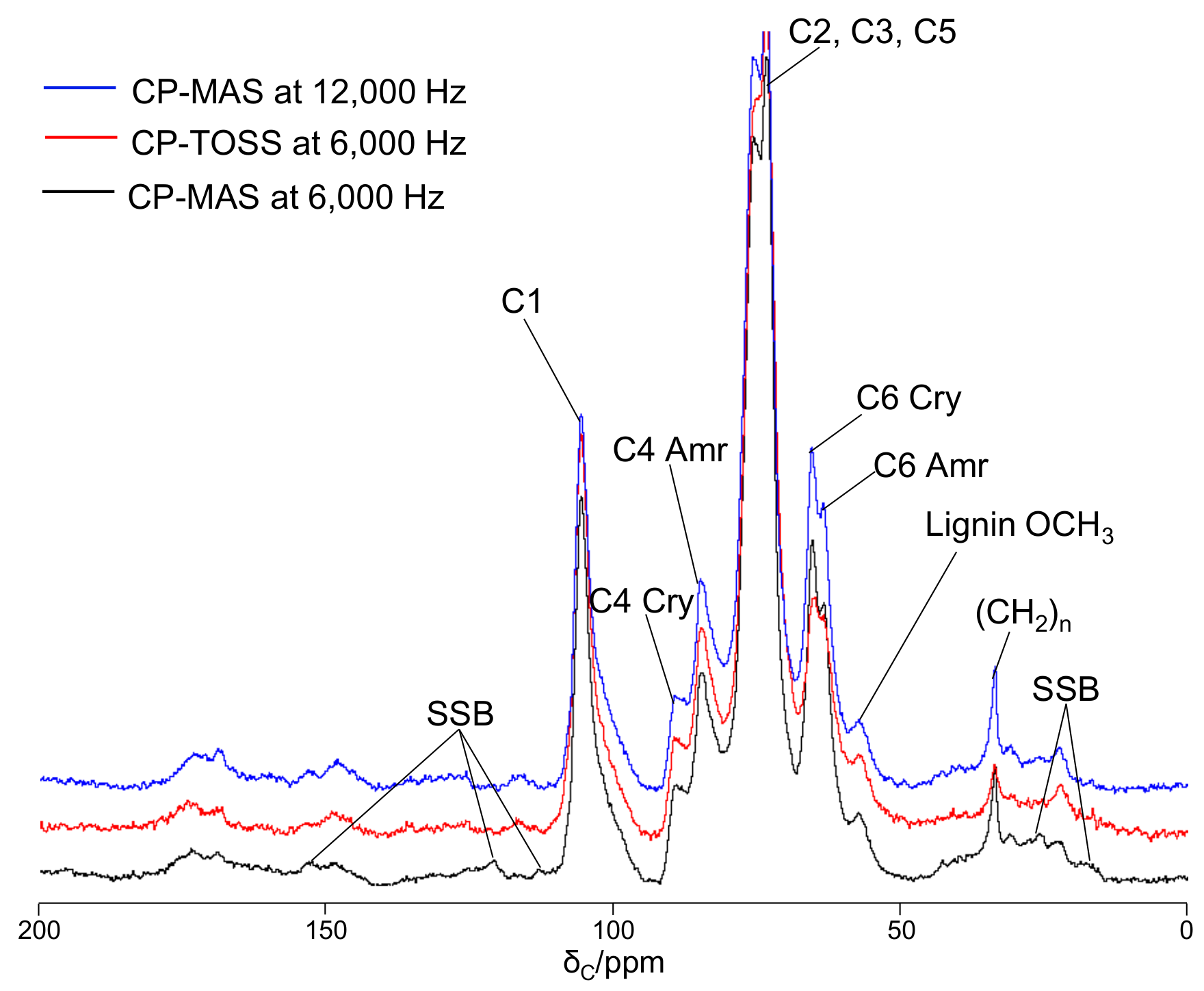

Supplement: Figure S2 — Comparisons of CP-MAS and CP-TOSS spectra using the spinning speed set to 6000 Hz and 12,000 Hz. CP-MAS spectra measured using the spinning speed set to 6000 Hz (black) and 12,000 Hz (blue) and CP-TOSS spectra measured using the spinning speed set to 6000 Hz (red) were obtained using the FM-processed sample. (TIF) [file pone.0066919.s002.tif]

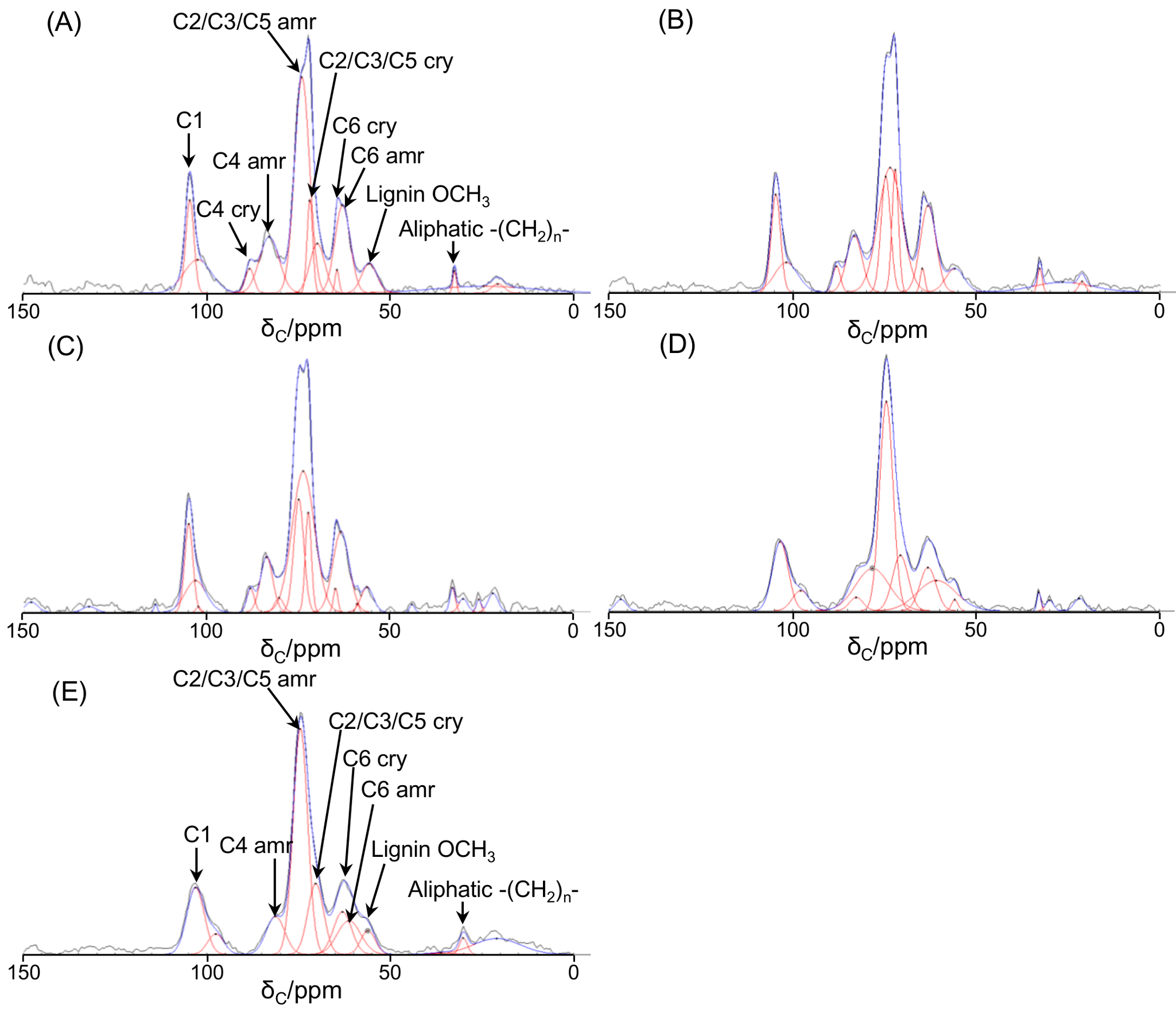

Supplement: Figure S3 — Peak separation of solid-state NMR using Fityk software. (A) FM-, (B) AM1-, (C) AM2-, (D) BM1-, and (E) BM2-processed samples. (TIF) [file pone.0066919.s003.tif]

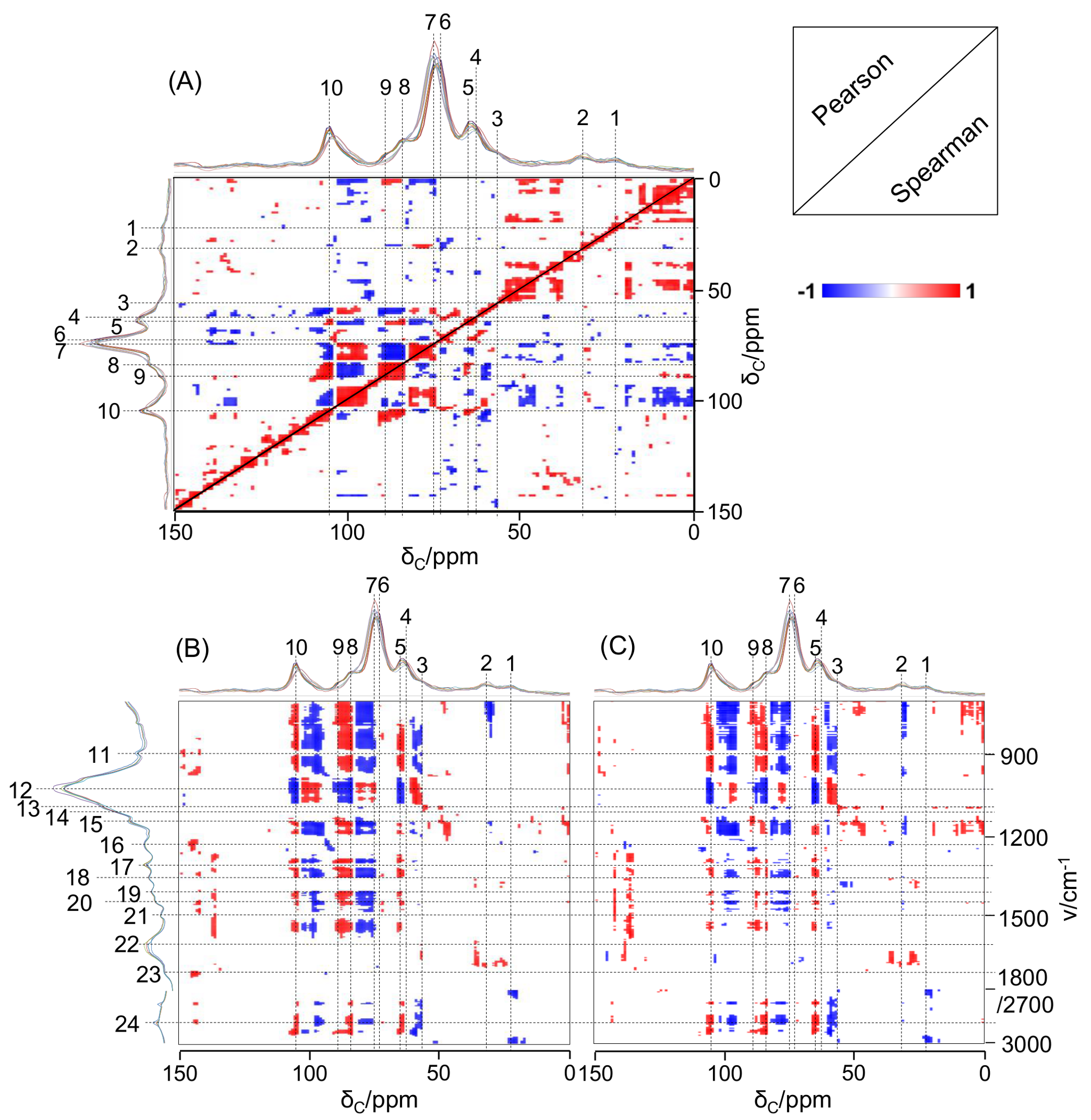

Supplement: Figure S4 — Homogeneous and heterogeneous correlation analysis of 13C-1H HETCOR and ATR-FTIR spectra. Homogeneous correlation heat map of NMR spectra (A) and heterogeneous correlation heat maps calculated by Pearson (B) and Spearman (C) between 13C-1H HETCOR and ATR-FTIR spectra. 1, CH3 in hemicellulose; 2, aliphatic –(CH2)n–; 3, OCH3 of lignin; 4, CH2OH of carbohydrates (C6 of amorphous cellulose); 5, CH2OH of carbohydrates (C6 of crystalline cellulose); 6 and 7, CHOH of carbohydrates (C2, C3, and C5 of cellulose); 8, CHOH of carbohydrates (C4 of amorphous cellulose); 9, CHOH of carbohydrates (C4 of crystalline cellulose); 10, OCHO of carbohydrates (C1 of cellulose); 11, COC vibration; 12, C–O stretching in cellulose and hemicellulose; 13, vibration of ester linkage; 14, aromatic skeletal and C–O stretching; 15, deformation vibrations of C–H bonds on benzene rings; 16, syringyl ring and C–O stretching in lignin and xylan; 17, C–H in cellulose and C1–O vibration in syringyl derivatives; 18, C–H deformation in cellulose and hemicellulose; 19, aromatic ring vibration; 20, asymmetric C–H bonding (in CH3 and –CH2–); 21, aromatic ring vibration; 22, stretching of C = O conjugated to aromatic rings; 23, stretching of C = O unconjugated to aromatic rings (oxidized side chains); 24, C–H stretching in cellulose. (TIF) [file pone.0066919.s004.tif]

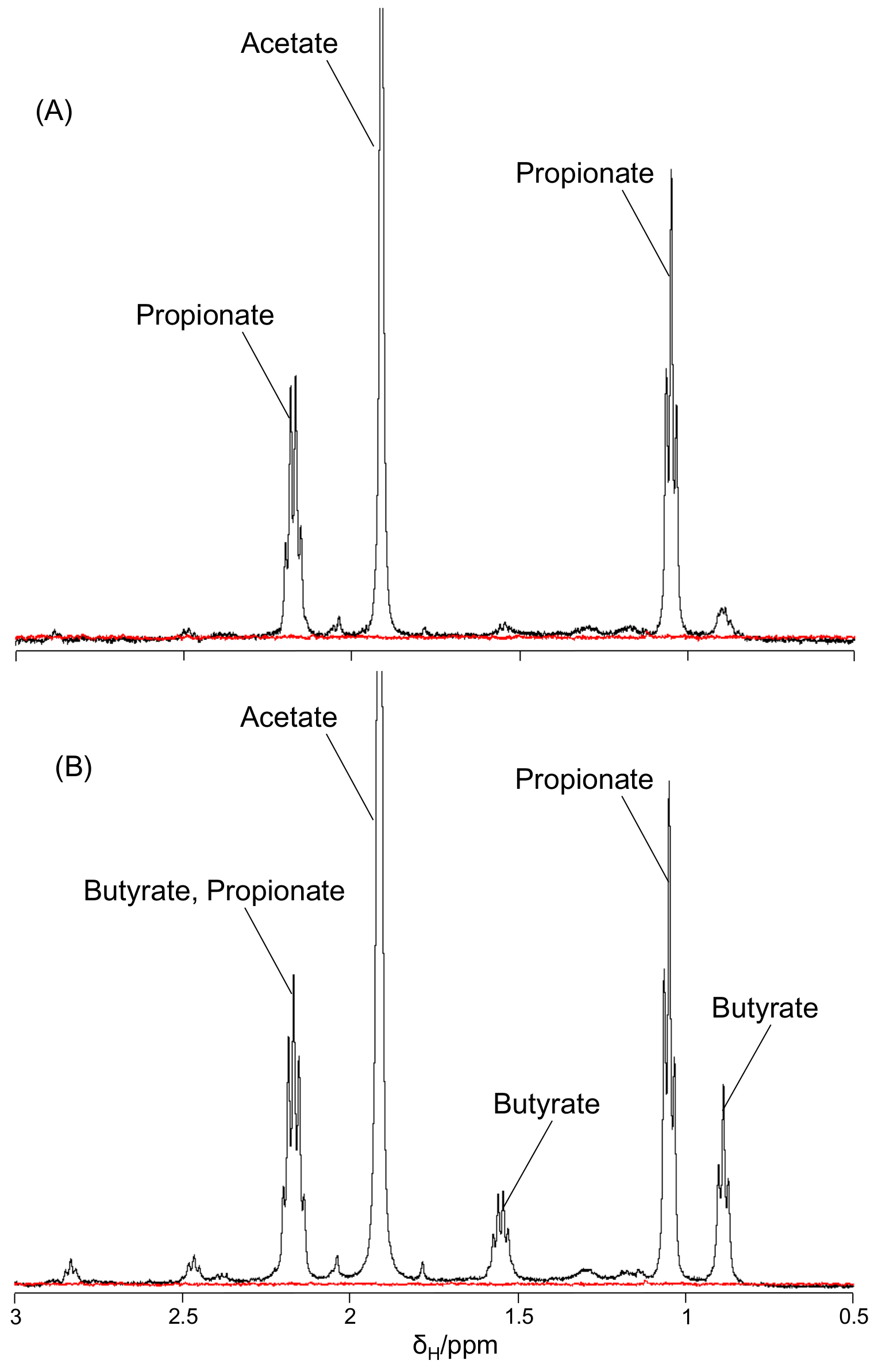

Supplement: Figure S5 — Comparison of 1H-NMR spectra of the control and FM- or BM2-processed incubation samples at Day 8. Red spectra are control and black spectra are FM- (A) and BM2-processed samples (B). (TIF) [file pone.0066919.s005.tif]

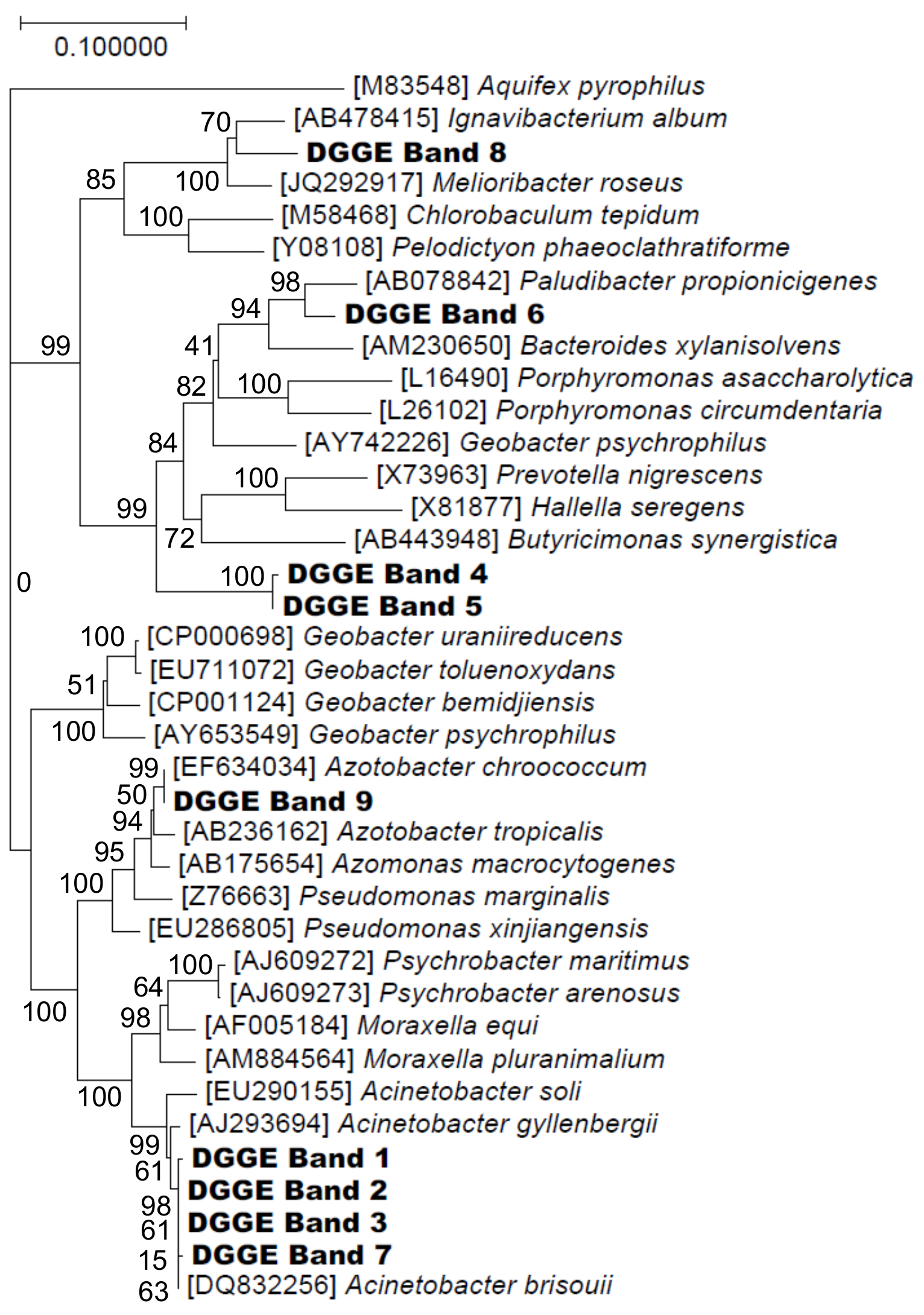

Supplement: Figure S9 — Phylogenetic tree constructed based on partial 16S rRNA gene sequences. The sequences determined in this study and those retrieved from the databases were aligned using CLUSTAL W2. The phylogenetic tree was then constructed using CLUSTAL W2 and Genetyx-tree software by the neighbor-joining method. The 16S rRNA gene fragment was amplified using the Univ954f and Univ1369r primer sets. The clones obtained were expressed as DGGE Bands 1–9. The 16S rRNA gene sequence of Aquifex pyrophilus [M83548] was used as an outgroup to root the tree. Indicated numbers in phylogenetic tree are bootstrap values. (TIF) [file pone.0066919.s009.tif]

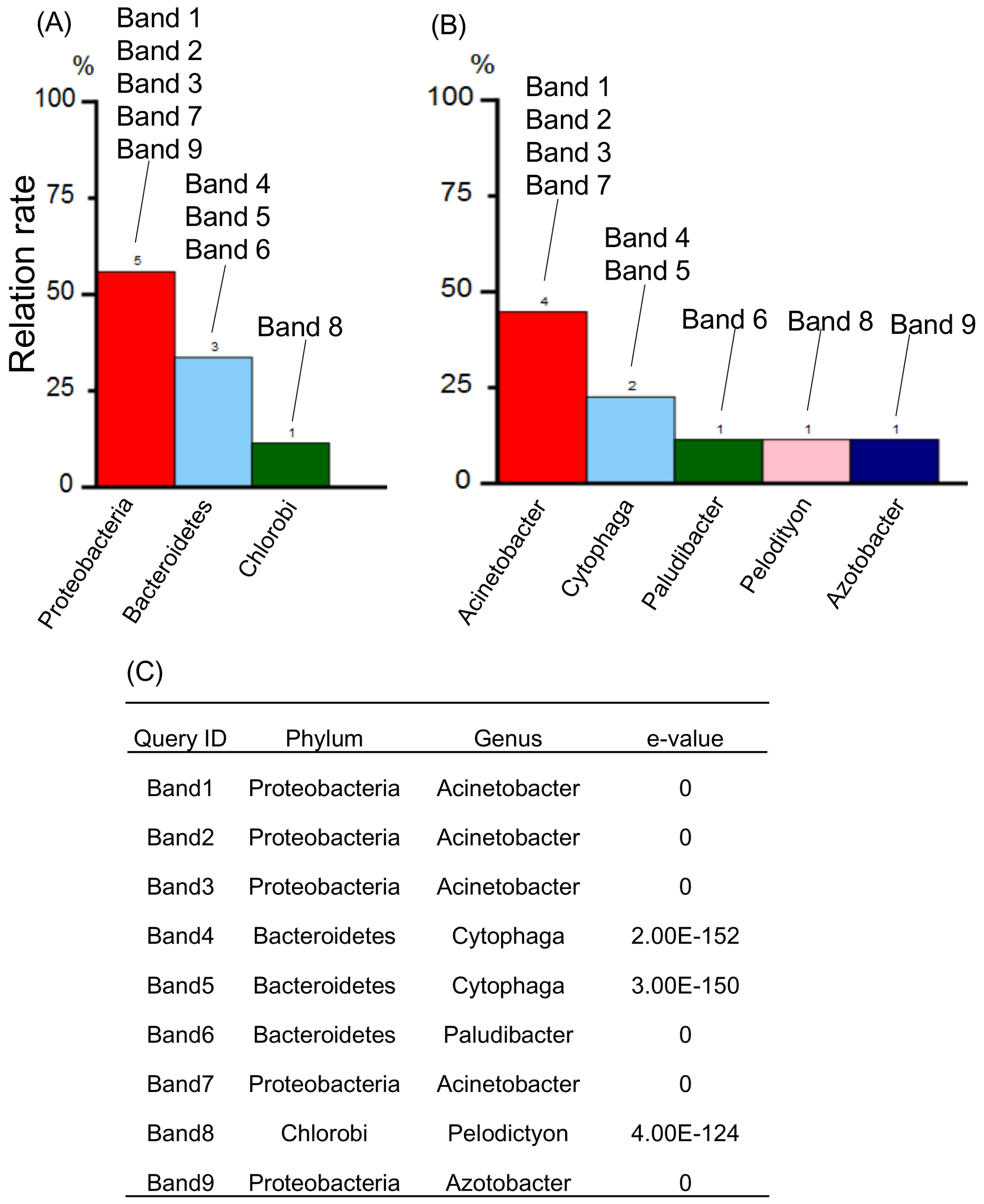

Supplement: Figure S10 — Classification of DGGE band sequences using E-class. Classification of each DGGE band sequence to the phylum (A) and genus level (B) and its summary (C). (TIF) [file pone.0066919.s010.tif]
