# Supplementary material for: Differences in Cellulosic Supramolecular Structure of Compositionally Similar Rice Straw Affect Biomass Metabolism by Paddy Soil Microbiota
Source: PLoS One. 2013 Jun 19;8(6):e66919. doi: 10.1371/journal.pone.0066919 (PMC3686774; doi:10.1371/journal.pone.0066919)
Supplement: Table S1 — The list of annotated metabolites in BM2 samples extracted by D2O solvent detected in the 1H-13C HSQC spectra. (DOCX) [file pone.0066919.s011.docx]

Table S1. The list of annotated metabolites in BM_2_ samples extracted by D_2_O solvent detected in the ^1^H-^13^C HSQC spectra.

| Components | Detection (%) | Peak No. |
| --- | --- | --- |
| Cellobiose | 68.4 | 1, 4, 7, 14, 15, 20, 21, 25, 26, 28, 29, 30 |
| D-Arabitol | 60.0 | 8, 16, 23 |
| D-Glucuronate | 66.7 | 1, 9, 11, 21, 24, 30 |
| D-Xylose | 70.0 | 2, 3, 10, 13, 18, 24, 27 |
| Ethylene glycol | 100.0 | 16 |
| Glycine | 100.0 | 22 |
| Maltose | 66.7 | 5, 6, 7, 9, 11, 12, 13, 17, 19, 20, 21, 24, 28, 30 |
